# Leveraging serum CHI3L1 and machine learning for non-invasive liver fibrosis diagnostics

**DOI:** 10.1016/j.isci.2026.117372

**Published:** 2026-08-31

**Authors:** Haizhen Chen, Gaixia Zhang, Yingwen Zhang, Li Li, Yuanyuan Che, Jing Huang

**Affiliations:** 1 Department of Laboratory Medicine, The First Hospital of Jilin University, Changchun, Jilin 130021, China; 2 Department of Clinical Laboratory, Xi’an Children’s Hospital, Affiliated Children’s Hospital of Xi’an Jiaotong University, Xi’an, Shaanxi 710003, China; 3 Department of Hepatology, The First Hospital of Jilin University, Changchun, Jilin 130021, China

**Keywords:** CHI3L1, liver fibrosis, machine learning, non-invasive

## Abstract

This study was aimed to develop non-invasive liver fibrosis diagnostic model using chitinase 3-like protein 1 (CHI3L1) based on machine learning (ML).1,011 follow-up patients (556 training/455 validation) were enrolled, staging fibrosis via transient elastography (F01, F2, F23, F34, and F4). Demographic, clinical, and laboratory parameters were collected. Fibrosis-related indicators were selected by differential analysis and correlation analysis. ML models were developed, optimized, and validated. Of 23 initial laboratory indicators, 15 indicators were selected to developed a 5-stage liver fibrosis diagnostic model (XG-Boost, FIB5-15, area under the curve [AUC] = 0.93, sensitivity = 0.70, specificity = 0.93). This was refined to a 7-indicator model (FIB5-7, area under the ROC curve [AUC] = 0.98). By merging F2/F23 and F34/F4, a 3-category optimized model (FIB3-7, AUC = 0.94) and a 2-category model (FIB2-7, AUC = 0.95) were created to identify reversible fibrosis. The final FIB2-7 model significantly outperformed aspartate aminotransferase‑to‑platelet ratio index (APRI) and FIB-4 (*p* < 0.001). The CHI3L1-related liver fibrosis models are applicable across multiple clinical scenarios.

## Highlights

• Establishment of CHI3L1-related liver fibrosis diagnosis model by machine learning
• Accurate and fast laboratory diagnosis method of liver fibrosis
• Sensitive, specific, and visual diagnosis model for multiple clinical scenarios

## Graphical abstract

**Figure:**
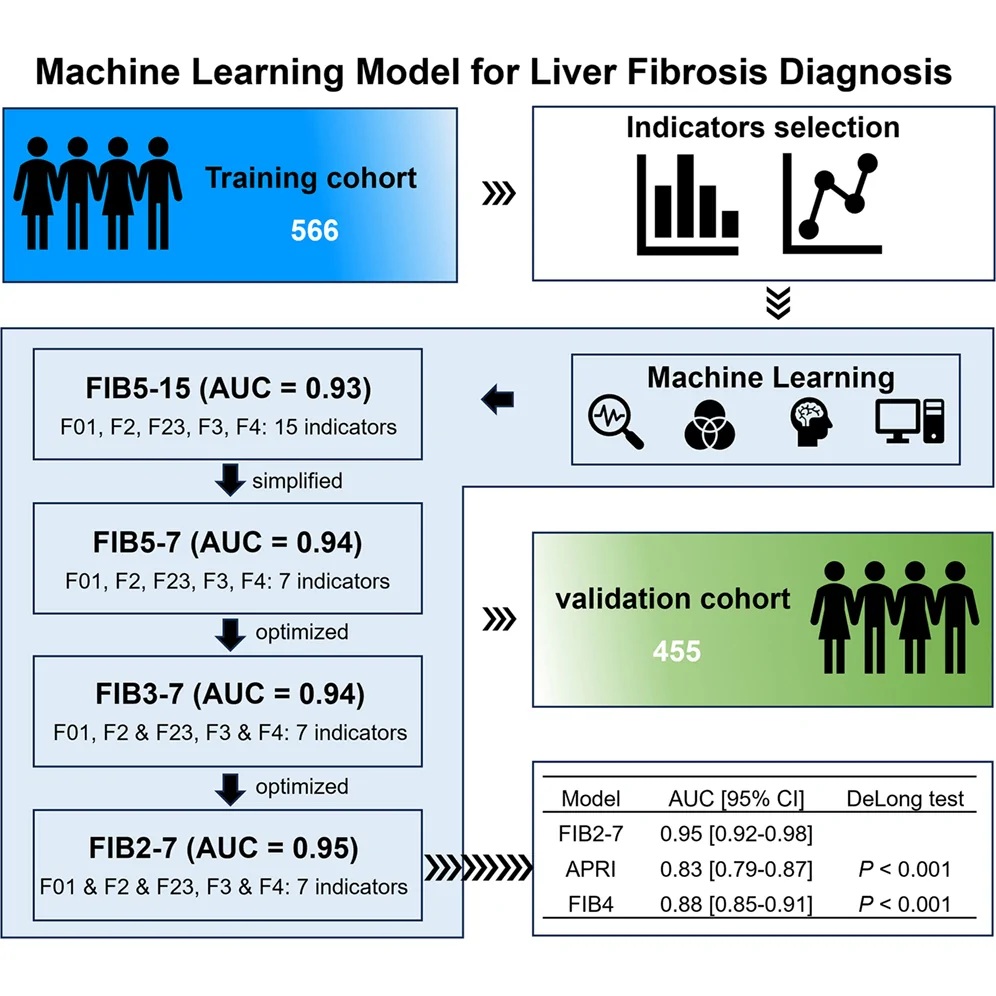

## Teaser

Health sciences; Diagnostics; Clinical measurement in health technology; Bioinformatics

## Introduction

Liver fibrosis is a crucial stage in the progression of liver diseases, where various factors such as inflammatory stimuli, oxidative stress, and hepatocellular damage activate hepatic stellate cells.^1^ These cells undergo activation and transform into myofibroblasts, leading to the accumulation of excessive extracellular matrix, resulting in liver scarring and liver fibrosis. The chances of reversing fibrosis significantly decrease once the degree of liver fibrosis reaches the advanced stage (F3 and F4 stages). Therefore, an early diagnosis and treatment of liver fibrosis can effectively reverse the progression of liver disease and potentially cure liver disorders. The gold standard for diagnosing liver fibrosis is liver tissue biopsy to determine the degree of fibrosis. However, liver biopsy has several disadvantages, such as invasive trauma, high cost, and the possibility of sampling errors or inappropriate tissue collection.^2^^,^^3^ Consequently, not all patients with liver disease are suitable candidates for liver biopsy, resulting in a continuous need to identify reliable and non-invasive diagnostic methods. Some non-invasive imaging techniques have been widely used in the diagnosis of liver fibrosis as a consequence of the advancement of medical imaging technologies. For example, transient elastography (TE) and magnetic resonance elastography assess the elasticity and stiffness of the liver, providing an indirect reflection of the degree of fibrosis.^4^^,^^5^^,^^6^^,^^7^ Systematic review analyses showed high accuracy of TE examination in screening and monitoring liver fibrosis, including alcoholic or non-alcoholic liver diseases and especially viral hepatitis, being characterized by non-invasiveness, simplicity, and relative affordability, making it a reliable tool.^8^^,^^9^^,^^10^^,^^11^ Research has recently discovered that serum markers accurately reflect the degree of liver fibrosis. For example, blood-based fibrosis-related proteins or algorithms, including chitinase 3-like protein 1 (CHI3L1),^12^ hyaluronic acid (HA), laminin (LN), type III procollagen N-terminal peptide (PIIINP), type IV collagen (Col IV),^13^^,^^14^^,^^15^ matrix metalloproteinases and their inhibitors,^16^^,^^17^ as well as inflammatory mediators, are measured in the serum to evaluate the degree of liver fibrosis. These methods are advantageous thanks to their non-invasiveness, convenience, and good repeatability, although further research and validation are still needed. Artificial intelligence (AI) technology, with its rapid development, is also widely applied in liver fibrosis diagnosis.^18^ AI uses machine learning (ML) and analysis of large amounts of clinical information, imaging data, and laboratory indicators to provide an accurate diagnosis and prediction of liver fibrosis.^19^ For instance, AI-based algorithms for imaging or serum analysis based on deep learning automate the grading of liver fibrosis. Thus, AI technology is becoming an essential tool in the diagnosis of liver fibrosis, although it requires extensive data validation and continuous improvement.

This study aimed to examine liver fibrosis serum marker CHI3L1 in a large clinical cohort and integrate it with TE technology through AI ML methods to establish a non-invasive liver fibrosis diagnostic model for various causes of liver disease.

## Results

### Clinical cohort baseline characteristics

**Figure 1:**
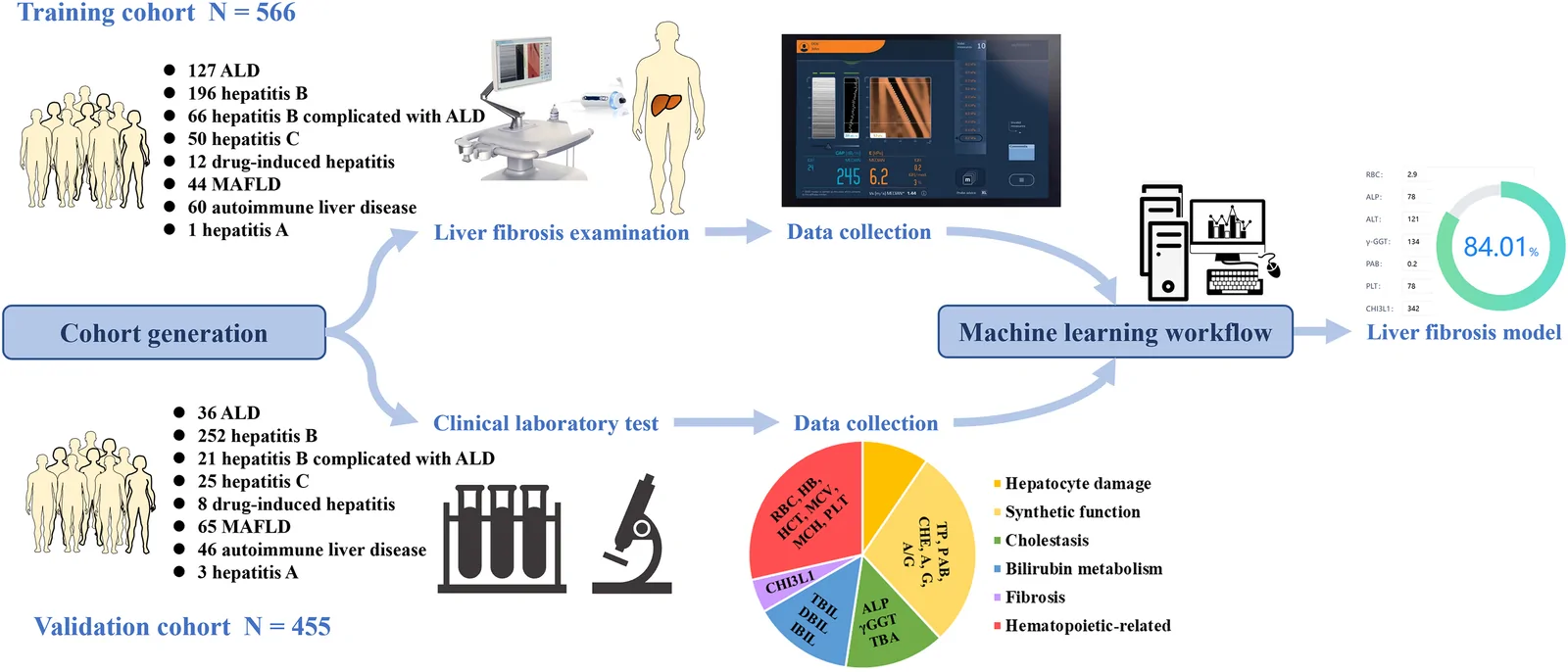
Study diagram ALD, alcohol-related liver disease; MAFLD, metabolic dysfunction-associated fatty liver disease.

This study included a total of 1,011 follow-up patients from a liver disease clinic, with 556 in the training cohort and 455 in the validation cohort (Figure 1). Clinical parameters of all participants were collected, for a total of 54 indicators. Indicators with a fill rate of less than 60% were excluded, resulting in 23 indicators being included in this study. Table 1 summarizes the baseline characteristics of participants with different stages of fibrosis in the training cohort. Apart from body mass index (BMI) (*p* = 0.095) and mean corpuscular volume (MCV) (*p* = 0.103), which were not significantly different among the five fibrosis stages (F01, F2, F23, F34, and F4) in the training cohort, the remaining 21 indicators were significantly different among fibrosis stage groups (*p* < 0.05).

**Table 1:** Baseline clinical characteristics

Baseline features	Training cohort					
	F01 (*n* = 186)	F2 (*n* = 87)	F23 (*n* = 72)	F34 (*n* = 80)	F4 (*n* = 131)	All (*n* = 556)
Sex	–	–	–	–	–	**∗∗**
Male	120 (64.5%)	51 (58.6%)	52 (72.2%)	55 (68.8%)	109 (83.2%)	387 (69.6%)
Female	66 (35.5%)	36 (41.4%)	20 (27.8%)	25 (31.2%)	22 (16.8%)	169 (30.4%)
Age (year)	45 (35–54)	51 (39–57)	52 (43–57)	54 (44–59)	52 (48–59)	50 (40–57) **∗∗**
BMI (kg/m^2^)	24.2 (22.0–26.4)	24.2 (22.5–26.3)	25.5 (23.7–27.7)	23.6 (21.2–26.0)	24.5 (22.5–27.3)	24.2 (22.1–27.0)
AST (U/L)	23.7 (20.2–29.9)	28.7 (22.0–41.0)	35.5 (27.8–53.3)	37.9 (28.6–56.3)	40.9 (28.4–71.5)	29.7 (22.8–46.4) **∗∗**
ALT (U/L)	23.3 (16.4–35.1)	25.6 (16.2–45.0)	36.7 (26.3–51.8)	35.0 (21.7–64.2)	28.7 (18.9–53.6)	28.5 (18.3–47.7) **∗∗**
γGGT (U/L)	27.9 (19.0–52.4)	41.0 (20.7–65.7)	55.0 (31.6–110.1)	59.4 (31.8–137.5)	59.0 (35.8–149.0)	42.7 (24.4–89.3) **∗∗**
ALP (U/L)	79.1 (66.5–98.6)	89.2 (69.7–118.9)	85.3 (72.0–124.6)	107.1 (86.0–130.5)	114.9 (82.8–143.8)	90.1 (71.8–120.4) **∗∗**
CHE (U/L)	8815 (7761–9982)	8155 (6749–9585)	8294 (6374–9940)	6728 (5227–8246)	3734 (2716–5217)	7552 (5287–9274) **∗∗**
TP (g/L)	76.5 (73.4, 78.8)	75.6 (72.5, 78.7)	76.1 (74.4, 79.6)	75.4 (72.2, 80.4)	63.0 (57.2, 72.8)	75.1 (69.9, 78.6) **∗∗**
ALB (g/L)	45.9 (44.1, 47.5)	44.5 (43.4, 47.0)	44.9 (42.8, 47.0)	42.8 (39.9, 45.5)	33.4 (28.1, 38.7)	44.1 (39.7, 46.5) **∗∗**
GLO (g/L)	30.5 (27.8, 32.6)	30.3 (28.4, 32.9)	30.5 (29.0, 35.6)	33.0 (29.7, 37.1)	31.2 (26.9, 36.2)	30.8 (28.0, 34.2) **∗∗**
A/G	1.52 (1.36–1.67)	1.49 (1.30–1.60)	1.47 (1.22–1.60)	1.28 (1.12–1.49)	1.10 (0.91–1.27)	1.42 (1.16–1.60) **∗∗**
TBIL (μmol/L)	15.3 (11.9–19.4)	14.7 (11.7–18.7)	18.9 (14.3–23.6)	19.9 (15.4–26.4)	30.0 (17.3–64.8)	17.4 (13.0–25.4) **∗∗**
DBIL (μmol/L)	2.6 (2.0–3.3)	2.4 (2.0–3.3)	3.5 (2.7–4.5)	3.8 (2.9–6.7)	8.4 (4.5–25.6)	3.3 (2.3–5.7) **∗∗**
IBIL (μmol/L)	12.6 (9.7–16.1)	12.4 (9.6–15.3)	15.6 (12.0–19.5)	15.6 (12.2–19.8)	21.2 (12.6–42.8)	13.9 (10.7–19.7) **∗∗**
TBA (μmol/L)	1.8 (1.3–3.1)	2.6 (1.6–4.8)	3.5 (2.3–12.3)	6.9 (4.4–15.5)	31.6 (13.9–94.8)	3.9 (1.8–15.0) **∗∗**
RBC (10^12^/L)	4.88 (4.49–5.31)	4.99 (4.42–5.43)	4.97 (4.48–5.30)	4.71 (4.40–5.05)	3.71 (3.00–4.30)	4.63 (3.93–5.09) **∗∗**
HB (g/L)	151 (130–161)	150 (137–163)	149 (135–165)	148 (138–160)	111 (93–136)	144 (119–159) **∗∗**
HCT	0.44 (0.40–0.47)	0.45 (0.41–0.48)	0.44 (0.41–0.49)	0.43 (0.43–0.46)	0.33 (0.28–0.40)	0.42 (0.35–0.46) **∗∗**
MCV (fL)	89.4 (87.1–92.8)	90.6 (87.6–93.3)	90.5 (87.6–93.3)	90.4 (88.0–96.4)	91.7 (86.5–98.3)	90.5 (87.2–94.5)
MCH (pg)	30.3 (29.2–31.6)	30.6 (29.4–31.8)	30.8 (29.5–31.9)	31.2 (30.1–32.6)	31.7 (29.6–34.3)	30.8 (29.4–32.5) **∗∗**
PLT (10^9^/L)	220 (215–227)	216 (216–216)	187 (156–205)	132 (124–146)	79 (52–107)	197 (125–220) **∗∗**
CHI3L1 (ng/mL)	45.8 (33.1–70.2)	52.0 (36.5–107.7)	70.8 (41.8–134.4)	99.7 (66.3–196.3)	184.3 (101.2–320.0)	74.84 (43.0–159.2) **∗∗**

The classification data of clinical characteristics are represented as the frequency (percentage), and the quantitative data are represented as median (interquartile range). In the first row of the table, “*n*” represents the number of participants in the liver fibrosis group. The Pearson’s chi-squared test was used for difference of sex between groups, and the independent Kruskal-Wallis *H* test was used for other baseline features. **∗**,*p* < 0.05; **∗∗**, *p* < 0.01. BMI, body mass index; AST, aspartate aminotransferase; ALT, alanine aminotransferase; γGGT, gamma-glutamyl transferase; ALP, alkaline phosphatase; CHE, cholinesterase; TP, total protein; ALB, albumin; GLO, globulin; A/G, ratio of albumin and globulin; TBIL, total bilirubin; DBIL, direct bilirubin; IBIL, indirect bilirubin; TBA, total bile acids; RBC, red blood cell count; HB, hemoglobin; HCT, hematocrit; MCV, mean corpuscular volume; MCH, mean corpuscular hemoglobin; PLT, platelet; CHI3L1, chitinase 3 like 1.

### Selection of fibrosis biomarkers

**Figure 2:**
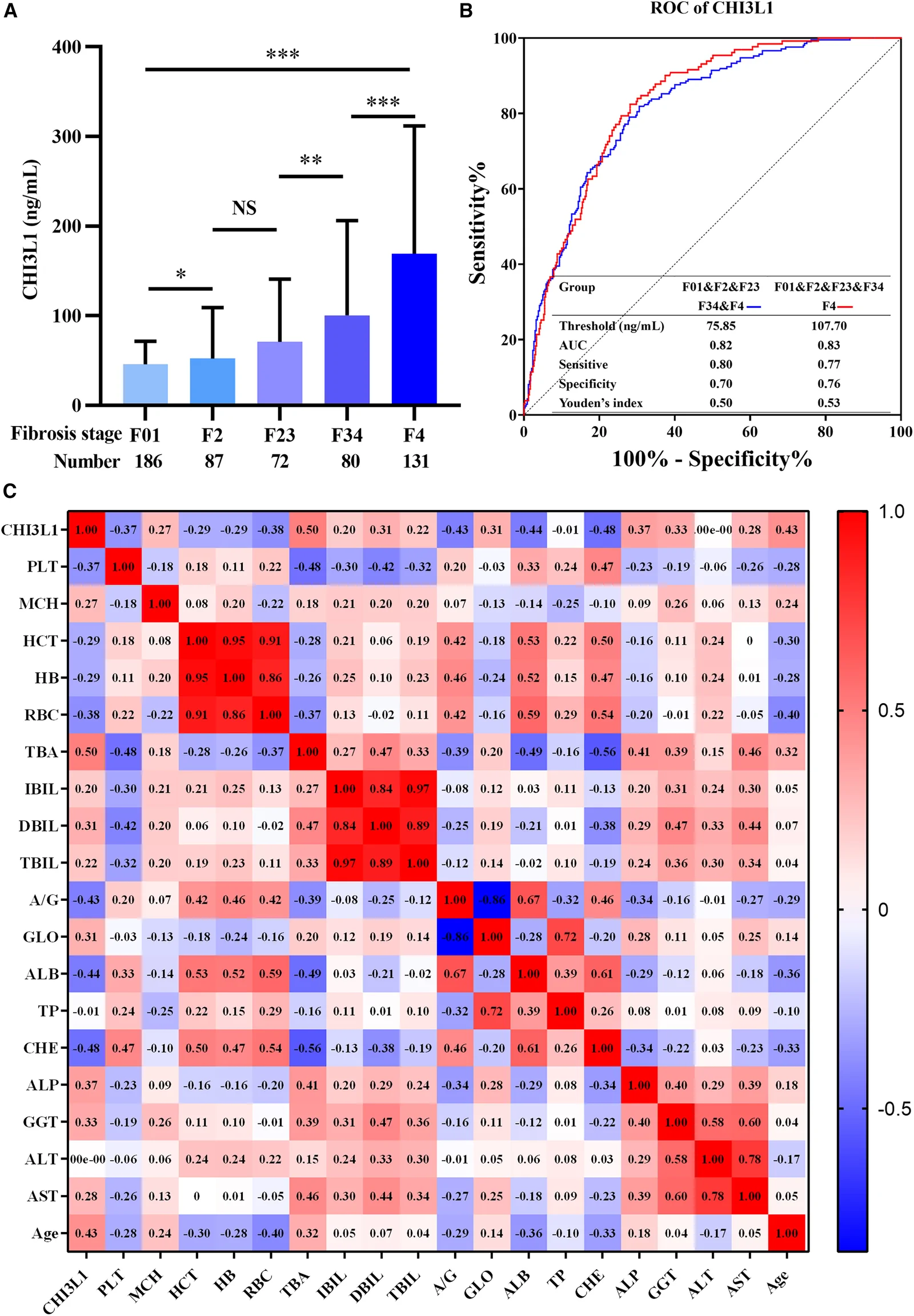
Screening of fibrosis markers (A) CHI3L1 level among different liver fibrosis groups. Data are represented as median with interquartile range. **∗***p* < 0.05, **∗∗***p* < 0.01, *∗*∗*∗p* < 0.001 by Mann-Whitney test between two groups. NS, no significance. Fibrosis stage F01, *n* = 186; F2, *n* = 87; F23, *n* = 72; F34, *n* = 80; and F4, *n* = 131. (B) ROC of CHI3L1 diagnosed for fibrosis progression (F34 and F4) and cirrhosis (F4). ROC analysis was performed based on the cut-off values of advanced liver fibrosis (78.85 ng/mL) and cirrhosis (107.70 ng/mL) in the CHI3L1 detection instruction manual. Fibrosis stage F01&F2&F23, *n* = 345; F34&F4, *n* = 211; F01&F2&F23&F34, *n* = 425; and F4, *n* = 131. (C) Association of 20 liver fibrosis-related clinical features. Spearman correlation analysis was performed on 20 numerical variables. The number is correlation coefficient, which was used to generate a heatmap.

The liver fibrosis-specific serum marker CHI3L1 included in this study was significantly different among the five fibrosis stages (Figure 2A, *p* < 0.001). The accuracy of CHI3L1 in diagnosing liver fibrosis was assessed by performing a receiver operating characteristic (ROC) analysis. The ability of CHI3L1 diagnosed for fibrosis progression (F34 and F4, area under the ROC curve [AUC] = 0.82, sensitivity = 0.80, specificity = 0.70) and cirrhosis (F4, AUC = 0.83, sensitivity = 0.77, specificity = 0.76) performed similarly, demonstrating that CHI3L1 could effectively discriminate severe stages of liver fibrosis, as shown in Figure 2B. However, in a single-marker analysis, Youden’s index was not ideal for distinguishing fibrosis progression at the designated cut-off threshold (Figure 2B). Therefore, a comprehensive analysis of multiple markers was required to explore the optimal combination of serum indicators for liver fibrosis stages. Spearman correlation analysis was performed on 20 numerical variables excluding sex (Figure 2C; Table S1) to avoid the overfitting of the model due to strong correlations among indicators, and variables with an absolute value of correlation coefficient R > 0.75 were excluded from the model construction (retaining a single variable). The results indicated a high correlation (with R > 0.75) among hematocrit (HCT), hemoglobin (HB), and red blood cell count (RBC) (R = 0.95, 0.91, and 0.86, respectively); indirect bilirubin (IBIL), direct bilirubin (DBIL), and total bilirubin (TBIL) (R = 0.84, 0.97, and 0.89, respectively); globulin (GLO) and ratio of albumin and globulin (A/G) (R = −0.86); and aspartate aminotransferase (AST) and alanine aminotransferase (ALT) (R = 0.78); thus, RBC, TBIL, GLO, and AST were finally used to develop the model.

### ML model establishment for liver fibrosis diagnosis

BMI, MCV, HCT, HB, IBIL, DBIL, A/G, and ALT were excluded after performing the differential and correlation analyses (Table 1; Figure 2C). Finally, a non-invasive diagnostic model of liver fibrosis with five classifications (named FIB5-15) was established using ML based on the 15 clinical indicators in the serum. The liver fibrosis model XG-Boost performed the best (area under ROC curve, AUC = 0.93, sensitivity = 0.70, specificity = 0.93) among the developed five liver fibrosis models (Table 2). The top 7 indicators with relative weights (> 0.35) were selected to simplify the analysis model and build an XG-Boost model again (named FIB5-7, Table 3; Figure S1, website see supplemental information), which also showed good diagnostic performance (AUC = 0.94, sensitivity = 0.67, specificity = 0.93) and no significant difference in inter-group AUC was found compared to the FIB5-15 analysis model (*p* > 0.05, Table S2). FIB5-7 demonstrated a good diagnostic value for no fibrosis (AUC = 0.96, sensitivity = 0.90, specificity = 0.83, Table 3) and cirrhosis (AUC = 0.98, sensitivity = 0.77, specificity = 0.95, Table 3). However, the sensitivity for diagnosing mild fibrosis stage (F2, F23, and F34; Table 4) was not ideal, although the specificity was high, indicating that the model had good diagnostic value for distinguishing the progression of liver fibrosis stages.

**Table 2:** The FIB5-15 liver fibrosis diagnosis models

Model	AUC	F1 score	Sensitivity	Specificity	Accuracy	NPV	PPV
Ada-Boost	0.80	0.51	0.53	0.90	0.63	0.92	0.66
XG-Boost	**0.93**	**0.72**	**0.70**	**0.93**	**0.75**	**0.94**	**0.74**
Random forest	0.90	0.69	0.67	0.93	0.73	0.93	0.74
Gradient boosting	0.86	0.57	0.56	0.91	0.65	0.92	0.61
Decision tree	0.85	0.68	0.67	0.92	0.69	0.92	0.71

The FIB5-15 liver fibrosis diagnosis model was established with the selected 15 indicators, including platelet, total bile acids, total protein, alkaline phosphatase, gamma-glutamyl transferase, red blood cell count, chitinase 3-like 1, cholinesterase, mean corpuscular hemoglobin, globulin, total bilirubin, albumin, aspartate aminotransferase, age, and sex. AUC, area under ROC curve; NPV, negative predictive value; PPV, positive predictive value. Bold indicates the best model.

**Table 3:** The FIB5-7 liver fibrosis diagnosis model of XG-Boost

Fibrosis stage	AUC	F1 score	Sensitivity	Specificity	Accuracy	NPV	PPV
F01	0.96	0.80	0.90	0.83	0.85	0.94	0.73
F2	0.91	0.59	0.50	0.96	0.89	0.91	0.72
F23	0.88	0.56	0.55	0.94	0.89	0.93	0.57
F34	0.94	0.64	0.63	0.94	0.90	0.94	0.65
F4	0.98	0.80	0.77	0.95	0.92	0.93	0.83
Mean	0.94	0.68	0.67	0.93	0.72	0.93	0.70

The FIB5-7 liver fibrosis diagnosis model was established with the selected top 7 relative weights indicators, including total bile acids, red blood cell count, alkaline phosphatase, gamma-glutamyl transferase, total protein, platelet, and chitinase 3-like 1. AUC, area under ROC curve; NPV, negative predictive value; PPV, positive predictive value.

**Table 4:** The FIB3-7 liver fibrosis diagnosis model of XG-Boost

Fibrosis stage	AUC	F1 score	Sensitivity	Specificity	Accuracy	NPV	PPV
F01	0.95	0.87	0.71	0.97	0.89	0.87	0.93
F2 & F23	0.89	0.70	0.77	0.82	0.81	0.90	0.64
F34 & F4	0.96	0.88	0.90	0.91	0.91	0.94	0.86
Mean	0.94	0.80	0.80	0.90	0.80	0.90	0.81

AUC, area under roc curve; NPV, negative predictive value; PPV, positive predictive value.

**Figure 3:**
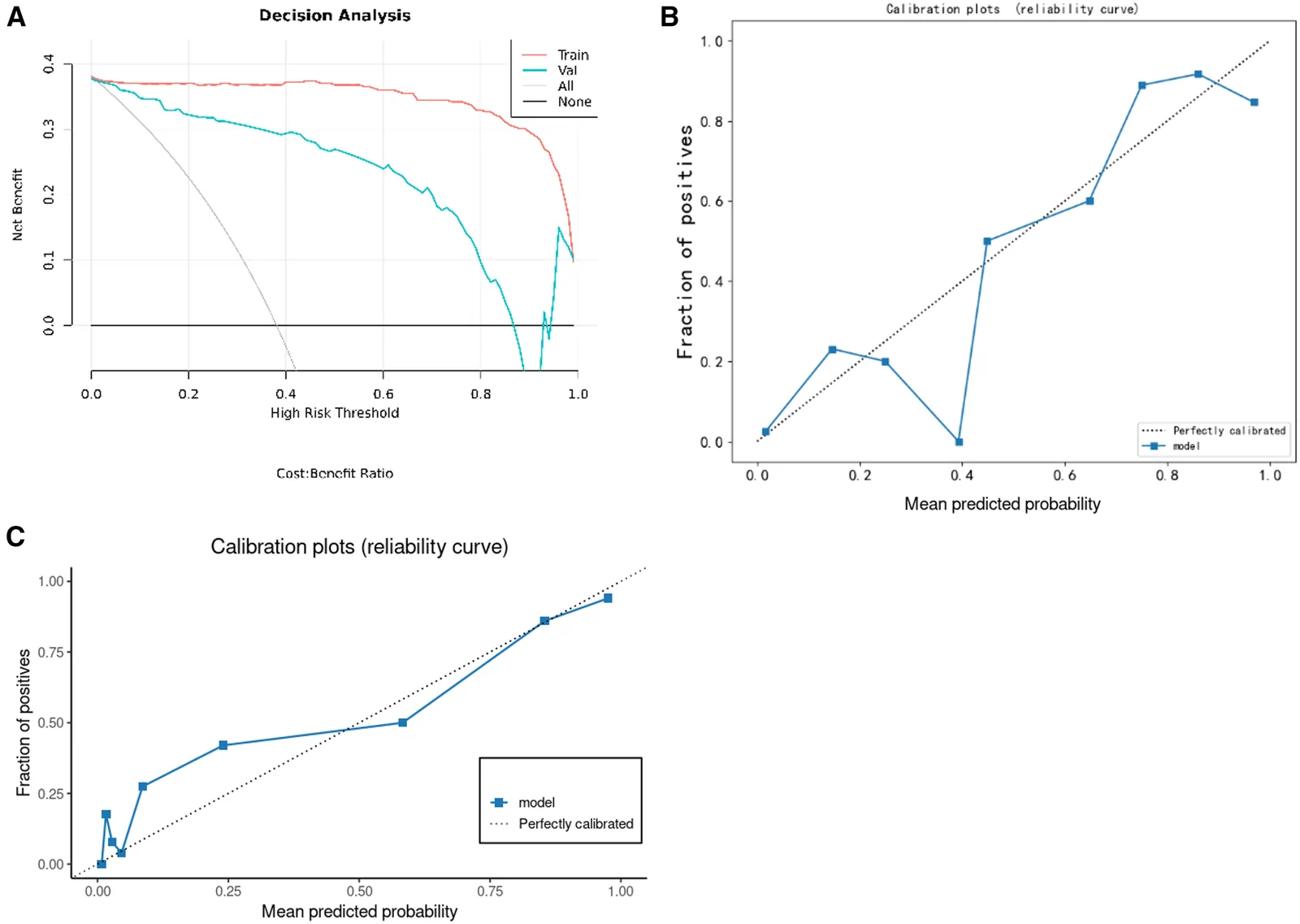
Decision curve and calibration curve analysis (A) Decision curve analysis of training cohort and validation cohort. (B) Calibration curve analysis of training cohort. (C) Calibration curve analysis of validation cohort.

The fibrosis stages were combined (F2 and F23, F34 and F4) to optimize the non-invasive liver fibrosis model and improve the diagnostic sensitivity. Subsequently, another XG-Boost model was built (named FIB3-7, website see supplemental information), and the results are shown in Table 4. FIB3-7 had an AUC of 0.94, with diagnostic sensitivity and specificity of 0.80 and 0.90, respectively (Figure S1). The XG-Boost for binary outcome was performed to demonstrate the diversity of the liver fibrosis model (named FIB2-7, website see supplemental information) with calibration curve and decision curve analysis. Binary classification was based on whether fibrosis was progressive, i.e., dividing five fibrosis stages into two groups: F0-F23 and F3-F4. The FIB2-7 model showed the best AUC (0.95, 95% confidence interval [CI]: 0.92–0.98), with sensitivity and specificity of 0.85 and 0.92, respectively (Table 5; Figure S1). The calibration curve and decision curve analysis of the FIB2-7 model showed excellent performance at any threshold, indicating higher benefit for patient intervention (TE or biopsy) compared to the intervention in all patients or no intervention at all (Figures 3A and 3B).

**Table 5:** The FIB2-7 liver fibrosis diagnosis model of XG-Boost

Positive stage	AUC	F1 score	Sensitivity	Specificity	Accuracy	NPV	PPV
F3-F4	0.95 (0.92–0.98)	0.90	0.85	0.92	0.86	0.90	0.87

AUC, area under ROC curve; NPV, negative predictive value; PPV, positive predictive value.

### External validation of the liver fibrosis diagnostic model

An external validation of the XG-Boost model was performed (*n* = 455; baseline clinical features shown in Table S3). Modeling validation was performed using FIB5-7 (FIB5-7 Val), which showed a slight decrease in AUC compared to the training cohort (AUC = 0.77, sensitivity = 0.48, specificity = 0.87; Table S4; Figure S1). The AUC increased to 0.82 (sensitivity = 0.68, specificity = 0.84; Table S5; Figure S1) after the optimization for 3-class classification (FIB3-7 Val). The XG-Boost model reached an AUC of 0.88 after the integration of liver fibrosis stages (FIB2-7 Val), with sensitivity and specificity of 0.67 and 0.92, respectively (Table S6; Figure S1). Similarly, calibration and decision curves showed an excellent performance (Figures 3A and 3C).

### Comparison of liver fibrosis diagnostic model with other models

The XG-Boost of FIB2-7 model was compared with the two models aspartate aminotransferase‑to‑platelet ratio index (APRI) and FIB-4, which are widely used in clinical diagnosis and treatment in the training cohort to verify its analytical performance. The results showed that FIB2-7 had higher and significantly different diagnostic efficacy (Table 6) compared to APRI and FIB-4.

**Table 6:** Comparison of the XG-Boost model FIB2-7, APRI and FIB-4

Model	AUC [95% CI]	Sensitive	Specificity	Best Youden’s index	*p* value	DeLong test
APRI	0.83 [0.79–0.87]	0.75	0.81	0.56	∗**∗∗**	**∗∗∗**
FIB4	0.88 [0.85–0.91]	0.81	0.83	0.63	**∗∗∗**	**∗∗∗**
FIB2-7	0.95 [0.92–0.98]	0.85	0.92	0.77	**∗∗∗**	–

*p* values are results of ROC analysis. The results of DeLong test are compared APRI and FIB-4 with FIB2-7. **∗∗∗**, *p* < 0.001.

## Discussion

Liver fibrosis is caused by the excessive accumulation and proliferation of fibrous connective tissue in the liver due to long-term inflammation and injury.^20^ Liver function is almost completely lost when liver fibrosis progresses to cirrhosis, leading to severe consequences such as liver failure and portal hypertension, significantly impacting human health. Liver fibrosis is a crucial precursor to liver cancer and is one of the significant risk factors for its development.^21^ Therefore, timely treatment and proper disease management can effectively alleviate the condition, slow disease progression, and improve the quality of life of patients already suffering from liver fibrosis. However, liver tissue pathological biopsy is not suitable to diagnose liver fibrosis in patients with mild liver fibrosis or medical institutions in small communities. When faced with a choice, patients are likely to choose a non-invasive and convenient diagnosis. Therefore, the development of a non-invasive diagnostic tool of liver fibrosis would be more practical for daily diagnosis and treatment processes, and clinical laboratories urgently need to develop more advanced and intelligent methods to solve this problem.

AI and ML have significant application value in the field of medical diagnosis. The combination of multiple indicators can improve the accuracy of clinical diagnosis, which is precisely the advantage of ML models. Many studies reported the establishment of ML diagnostic models through the joint use of multiple indicators, which demonstrate superior diagnostic value compared to the diagnosis using a single indicator of liver fibrosis.^22^^,^^23^^,^^24^ Zhang et al.^25^ built a non-invasive diagnostic model of liver fibrosis for chronic hepatitis B using five selected features including hepatitis B virus DNA (HBV-DNA), international normalized ratio, thrombin time, albumin (ALB), and platelet (PLT), and found that the decision tree model showed the best diagnostic performance among different independent datasets, with a mean AUC of 0.8617 (SD 0.02377) and relative standard deviation calculated based on the AUC values between groups at different fibrosis stages of 2.7585, respectively. Then, the decision tree model was applied to the training cohort and the external validation cohort, with the relative standard deviation of 0.116, showed more stable and the best generalized performance. Since our study exclusively enrolled patients from the hepatology outpatient follow-up cohort, coagulation tests are not routinely performed, only patients at F4 stage undergo routine coagulation testing. Consequently, we are unable to directly compare the diagnostic model of Zhang et al. with the model in this study. Another study from Canada by Sarvestany et al.^26^ developed an ensemble (ENS) algorithm by integrating five ML models using 12 parameters, achieving a mean area under the receiver operating characteristic curve (AUROC) of 0.795 (SD 0.031) and a mean AUROC of 0.787 (SD 0.042) without indeterminate classifications, outperforming five other ML models, APRI, and FIB-4. Interestingly, in one external validation cohort, the ENS (cutoff of 0.465 for F012 vs. F34) demonstrated a sensitivity of 80.8%, compared to 92.3% for TE (cutoff of 8.0 for F012 vs. F34), with both methods having the same specificity of 63.8%. Additionally, the AUROC of ENS (0.465) was only 0.053 lower than that of TE (8.0) (0.773 [95% CI: 0.699–0.834] vs. 0.826 [95% CI: 0.758–0.889]). This suggests that TE may provide accurate fibrosis diagnosis for most patients meeting the examination criteria, supporting the feasibility of using TE as the diagnostic standard for liver fibrosis assessment in outpatient follow-up patients in our study. Clinical laboratory data are comprehensive and rich, and a report not only reflects the numerical changes of test items but also more accurately indicates the risk of diseases, which was the starting point of our research.

CHI3L1, initially discovered in 1992 by Johansen et al. in the human osteosarcoma cell line MG63, belongs to the 18-glycosyl hydrolase family, with a peptide chain consisting of 383 amino acids and a relative molecular mass of approximately 40 kDa. Therefore, CHI3L1 is also known as YKL-40.^27^^,^^28^ *CHI3L1* gene expression in the liver is higher than in other tissues such as the heart, brain, and kidney, with the highest expression in hepatocytes and hepatic stellate cells.^29^ Therefore, as a new inflammatory marker and cytokine, CHI3L1 is a highly expressed protein in the liver and may be a good marker for liver diseases. Some studies demonstrated the potential role of CHI3L1 in liver fibrosis. CHI3L1 expression in patients with viral hepatitis is significantly increased in severely fibrotic liver tissue, and its level is correlated with the progression of fibrosis in viral hepatitis, indicating that CHI3L1 may be involved in the development of liver fibrosis.^30^^,^^31^ Moreover, CHI3L1 has a superior diagnostic value compared to HA and PIIINP.^32^^,^^33^ Harrison SA et al.^34^ used a prospective multicenter study to validate that CHI3L1 in non-alcoholic liver disease in combination with other blood markers (miR-34a-5p, alpha-2 macroglobulin, and glycated HB), non-invasively diagnoses non-alcoholic steatohepatitis and liver fibrosis (AUROC = 0.80, 95% CI: 0.73–0.85), and does not require adjustment for age, sex, BMI, or aminotransferase concentrations. CHI3L1 expression in alcoholic liver disease is also associated with cirrhosis, and higher CHI3L1 expression is correlated with shorter survival.^35^ Despite these observations, the exact mechanism of action of CHI3L1 in liver fibrosis remains unclear, requiring further research. Additionally, it is not yet known whether CHI3L1 can serve as a potential therapeutic target for liver fibrosis, thus needing further investigation.

In this study, various liver fibrosis diagnostic models were established using ML methods. The FIB5-7 liver fibrosis diagnostic model was established to comprehensively reflect liver function while reducing patient laboratory testing. The FIB3-7 and FIB2-7 models were established to improve the sensitivity of early fibrosis diagnosis, further increasing the diagnostic AUC and sensitivity. Different diagnostic models can be applied to different scenarios. FIB5-7 can be applied in community medical institutions and physical examination centers for the general population and patients with mild liver dysfunction. Patients with more specific liver diseases in large comprehensive medical institutions, such as hepatitis virus, alcohol, abnormal fat metabolism, and autoimmune disorders, can be more sensitively diagnosed with FIB3-7 and FIB2-7 for mild and advanced liver fibrosis, encouraging patients to seek timely treatment and relief. Indeed, diagnostic thresholds for liver fibrosis vary across different hepatic diseases.^36^^,^^37^^,^^38^ Certain liver conditions accompanied by acute hepatic inflammation (characterized by dramatic elevations in ALT and AST), such as acute viral hepatitis, drug-induced hepatitis, and alcoholic hepatitis, may yield exaggerated liver stiffness measurements (LSM) that do not accurately reflect the true fibrosis stage. Importantly, our study cohort exclusively comprised follow-up patients who had been rigorously screened through comprehensive medical record review to exclude those in acute hepatitis phases or with significant cholestasis. Furthermore, as highlighted in clinical guidelines, the natural age-related decline in ALT levels may lead to overestimation of fibrosis when applying age-dependent diagnostic models, particularly in elderly populations.^39^ During biomarker selection, we identified strong collinearity between AST and ALT across fibrosis stages (R = 0.78). In developing the FIB5-15 model, we retained AST while excluding ALT to mitigate inflammation-related bias. Subsequent refinement yielded the FIB5-7 model without AST incorporation, as its relative weighting proved insignificant (<0.35). This systematic modeling strategy effectively minimizes the confounding impact of hepatic inflammation on fibrosis assessment. Moreover, the visualization of model diagnosis results could also be presented directly and concisely to doctors and patients with higher acceptability.

This study used ML methods to comprehensively analyze clinical laboratory data, evaluated the diagnostic value of CHI3L1 as a new liver fibrosis biomarker, and established a non-invasive liver fibrosis diagnostic model with good accuracy and reliability. This model provided a comprehensive liver fibrosis assessment in clinical practice, enabling earlier and more effective interventions and treatments for patients with liver fibrosis.

### Limitations of the study

Our research also has some limitations. This study was performed in a single center, and although a single TE examiner reduced the impact of inconsistent TE results, it might also limit the ability of our diagnostic model to demonstrate similar excellent diagnostic value when applied to other centers. Therefore, our future plan is to validate this model in other centers and optimize it in larger cohorts. Additionally, this study did not use histopathological biopsy results as the diagnostic standard for liver fibrosis, which could introduce inaccuracies into the study. The premise of this study was to evaluate the diagnostic value of the CHI3L1 diagnostic model for liver fibrosis in patients examined in routine clinical practice, who typically do not require histopathological biopsies for diagnostic and treatment purposes. Furthermore, this study indicated that TE examination had a very high diagnostic accuracy in detecting liver fibrosis and could serve as a replacement for liver tissue biopsy as an initial screening test for liver fibrosis classification. Therefore, TE examination results were used as the standard for liver fibrosis classification.

## Resource availability

### Lead contact

Requests for further information and resources should be directed to and will be fulfilled by the lead contact, Jing Huang (huangj@jlu.edu.cn).

### Materials availability

This study did not generate new unique reagents.

### Data and code availability

• All data reported in this paper will be shared by the lead contact upon request. The raw experimental data have been archived in the research center’s institutional repository. The dataset cannot be publicly shared due to patient privacy data restrictions. Based on the establishment of a secure data sharing protocol and a joint learning framework, the data may be made available upon request with the approval of the corresponding author. Researchers interested in accessing the primary data may contact the corresponding author via email. Upon reasonable request and with appropriate justification, the authors will share the relevant datasets.
• This paper does not report original code. In the results section, we have provided an open access website for the diagnostic model. Please note that the indicators and results on the website are currently displayed in Chinese. To facilitate broader usability of the model, we recommend that readers use the Microsoft Edge browser to access the website. By utilizing the built-in translation feature of Microsoft Edge, the content can be automatically translated into the reader’s native language, thereby enhancing comprehension and usability for a wider audience. FIB5-7 website: https://dxonline.deepwise.com/prediction/index.html?baseUrl=%2Fapi%2F&id=41649&topicName=undefined&from=share&platformType=wisdom. FIB3-7 website: https://dxonline.deepwise.com/prediction/index.html?baseUrl=%2Fapi%2F&id=41668&topicName=undefined&from=share&platformType=wisdom. FIB2-7 website: https://dxonline.deepwise.com/prediction/index.html?baseUrl=%2Fapi%2F&id=41679&topicName=undefined&from=share&platformType=wisdom
• Any additional information required to reanalyze the data reported in this paper is available from the lead contact upon request.

## Acknowledgments

We want to thank all the patients who participated in this study for their cooperation and contributions. We sincerely thank Hao Zhang, Yuming Cheng, and Xialin Wang, data scientists at Beckman Coulter, for their help and suggestions in screening biomarkers and data analysis. This work is supported by Extreme Smart Analysis platform (https://www.xsmartanalysis.com/). This work was supported by the Science and Technology Capacity Enhancement Project of Jilin Provincial Health Commission (no. 2022JC059).

## Author contributions

All authors contributed to the study conception and design. Material preparation and experimental operation were performed by H.C., G.Z., Y.Z., L.L., and Y.C.; data collection and analysis were performed by G.Z. and H.C. The first draft of the manuscript was written by G.Z. and H.C. Experiment supervision and manuscript review were performed by J.H.

## Declaration of interests

The authors declare no competing interests.

## STAR★Methods

### Key resources table

**Table:** 

REAGENT or RESOURCE	SOURCE	IDENTIFIER
**Biological samples**		

Serum from follow-up patients	The First Hospital of Jilin University	N/A

**Deposited data**		

Raw and analyzed data	This paper	See Tables 1 and S3

**Software and algorithms**		

Extreme Smart Analysis platform	Beckman Coulter	https://www.xsmartanalysis.com/
GraphPad Prism (version 8.0.2)	GraphPad Software	https://www.graphpad.com/resources

**Other**		

Transient elastography	Echosens	FibroScan 502
Serum CHI3L1 level	YHLO Corporation	iFlash
Blood routine indexes	Sysmex Corporation	Sysmex XN-9000
Blood biochemical indexes	Beckman Coulter, Inc	AU5800
Coagulation function indexes	Sysmex Corporation	Sysmex cs-5100
Tumor markers	Roche Corporation	Cobas e601

### Experimental model and study participant details

This retrospective study was approved by the Ethics Committee of the First Hospital of Jilin University (NO. 2022-354). Follow-up patients (1,011 individuals from the population of Northeast China) who visited the Hepatology Clinic at the First Hospital of Jilin University between June 2021 and October 2022 were included in this study, and their TE test results were used as diagnostic criteria for liver fibrosis.

A total of 556 follow-up patients were included in the training cohort between June 2021 and August 2022. The inclusion criteria were the following: show up to the TE clinic for TE examination, and meet the TE examination criteria; history of viral hepatitis, alcoholic liver disease, non-alcoholic fatty liver disease, autoimmune liver disease and other liver diseases after inquiries or evidence of relevant laboratory examinations; laboratory tests were performed and serum was collected. The exclusion criteria were the following: not meeting the TE examination criteria, including presence of ascites, eating (fasting or 2-h postprandial test), severe obesity, narrow intercostal space, and cholestasis; being subjected to TE examination in the past six months and continuing to receive liver fibrosis treatment; comorbidity with other diseases, including hepatocellular carcinoma and history of hereditary diseases; special populations, such as pregnant women. A time-series independent method was used to establish training and validation sets, and 455 follow-up patients also from the First Hospital of Jilin University were included in the external validation cohort between September and October 2022. These patients were included for external validation purposes, undergoing the same diagnostic protocols as those applied in the training cohort for comparative analysis and assessment. In the training cohort of 556 follow-up patients, including 387 males (69.6%) and 169 females (30.4%), and the validation cohort of 455 follow-up patients, including 262 males (57.6%) and 193 females (42.4%), an analysis of differences between fibrosis groups using *Pearson’s Chi-squared* test revealed a significant difference in sex among the fibrosis groups in the training cohort (*p* < 0.01, Table 1), whereas no significant difference was observed in the validation cohort (*p* = 0.567, Table S3). In the training cohort, the established liver fibrosis diagnostic model FIB5-15 initially included sex as an indicator. However, after optimizing the model by reducing indicators, sex was excluded from the FIB5-7 liver fibrosis diagnostic model for low relative weight (0.0425). Consequently, the sex indicator, which showed no significant difference between fibrosis groups in the validation cohort, did not affect the validation of the fibrosis model. This study included biological sex as a clinical variable for statistical analysis. Due to limitations in patient privacy concerns and medical record collection, gender ancestry, race, and ethnicity were not analyzed, which may introduce certain limitations to the findings. Physicians performing TE examinations in this study received standardized training and had at least 500 instances of testing experience, demonstrating the ability to independently perform examinations.

### Method details

Serum samples of all subjects were collected, and the chemiluminescence method (Shenzhen YHLO Biotech Co., Ltd.) was used to detect the liver fibrosis protein CHI3L1. Demographic, clinical, and laboratory parameters of the subjects, including sex, age, BMI, clinical diagnosis, laboratory test results (including blood routine, liver function tests and tumor markers) were collected by a laboratory information system.

#### Machine learning platform and model development workflow

All statistical analyses and ML model construction were performed using the Extreme Smart Analysis platform (Beckman Coulter, https://www.xsmartanalysis.com/), a comprehensive R language-based environment specifically designed for clinical data mining and predictive modeling. The platform provides an integrated six-step workflow for predictive model development, encompassing data preprocessing, statistical analysis, feature selection, model training, performance evaluation, and external validation.

**Step 1: Liver fibrosis staging.** The study cohort was stratified into five groups based on TE-derived liver stiffness measurements (LSM) using the cut-off values routinely applied in our center’s FibroScan platform. Liver fibrosis was classified into five grades as follows: LSM <7.3 kPa was defined as F0–F1 stage (no or mild fibrosis); LSM 7.3–9.7 kPa as F2 (mild fibrosis); LSM 9.7–12.4 kPa as F23 (moderate fibrosis); LSM 12.4–17.5 kPa as F34 (severe fibrosis); and LSM >17.5 kPa as F4 (cirrhosis). For model optimization purposes, F34 was considered the threshold for irreversible or advanced fibrosis, while F23 served as the borderline for combining adjacent groups to enhance diagnostic sensitivity for clinically actionable cut-offs.
**Step 2: Feature selection and dimensionality reduction.** The independent Kruskal–Wallis H test was applied to assess the statistical differences of all 23 clinical features across the five fibrosis stages, and indicators showing significant differences (P < 0.05) were selected as candidate predictors. To avoid multicollinearity and overfitting, Spearman’s rank correlation analysis was performed on all numerical variables. When two or more variables exhibited a correlation coefficient |R| > 0.75, only the variable with the most established clinical relevance to fibrosis was retained, while the redundant ones were excluded. Specifically, RBC was retained over HB and HCT; TBIL was retained over IBIL and DBIL; GLO was retained over A/G; and AST was retained over ALT. This filtering process yielded a final set of 15 core features for model construction, comprising platelet count, total bile acids, total protein, alkaline phosphatase, gamma-glutamyl transferase, red blood cell count, CHI3L1, cholinesterase, mean corpuscular hemoglobin, globulin, total bilirubin, albumin, aspartate aminotransferase, age, and sex.
**Step 3: Model training and algorithm comparison.** Five machine learning classification algorithms were trained and compared using the 15 selected features in the training cohort: AdaBoost, XGBoost, Random Forest, Gradient Boosting Machine, and Decision Tree. All models were developed for five-class classification (F01, F2, F23, F34, and F4) using a 3- to 7-fold cross-validation strategy with random sample splitting within the training cohort. Model performance was evaluated using receiver operating characteristic (ROC) curves, and the area under the curve (AUC) was calculated for each model to assess discriminative ability.
**Step 4: Model selection and optimization.** The XGBoost algorithm, which demonstrated the highest overall AUC (0.93) and the most balanced sensitivity and specificity across fibrosis stages, was selected as the optimal model. Feature importance was ranked using the built-in gain-based importance metric, and the top seven indicators with relative weights exceeding 0.35 were retained to construct a simplified model (FIB5-7). To further improve diagnostic sensitivity for early-stage fibrosis, the five groups were consolidated into three categories (F01, F2–F23, and F34–F4) and a binary outcome (F0–F23 versus F3–F4) based on the clinical concept of fibrosis reversibility. Calibration curves were used to assess the agreement between model-predicted probabilities and observed outcomes, and decision curve analysis was performed to evaluate the net clinical benefit of the optimized model across a range of threshold probabilities.
**Step 5: External validation.** The temporally independent validation cohort (n = 455) was used to externally validate the optimized XGBoost models (FIB5-7, FIB3-7, and FIB2-7) without any model recalibration, preserving the original coefficients and cut-offs derived from the training set. Performance metrics, including AUC, sensitivity, specificity, accuracy, F1 score, and predictive values, were recalculated in the validation set for each model variant.
**Step 6: Model deployment.** Finally, the established diagnostic models were made publicly accessible as open online tools, enabling real-time non-invasive liver fibrosis risk assessment for clinical application. The web-based interfaces allow users to input the required laboratory parameters and immediately obtain the predicted fibrosis stage with corresponding probability estimates.

### Quantification and statistical analysis

#### Differential analysis

In the Extreme Smart Analysis platform, the independent Kruskal-Wallis H test was used to analyze differences in clinical indicators represented by median (interquartile range) between fibrosis groups in the training cohort (F01 *n* = 186, F2 *n* = 87, F23 *n* = 72, F34 *n* = 80, F4 *n* = 131, all *n* = 556) and external validation cohort (F01 *n* = 126, F2 *n* = 86, F23 *n* = 78, F34 *n* = 67, F4 *n* = 98, all *n* = 455). The Pearson’s Chi-squared test was used for difference of sex is represented as the frequency (percentage) between groups (Tables 1 and S1). Using GraphPad Prism (version 8.0.2) to analyze the differences by Mann-Whitney test in serum CHI3L1 levels among liver fibrosis groups (Figure 1A, CHI3L1 levels are represented as median with interquartile range). The Delong test was utilized on the Extreme Smart Analysis platform to compare the performance differences between the constructed non-invasive liver fibrosis diagnostic models (FIB5-15 vs. FIB5-7, Table S2).

#### Correlation analysis

The spearman correlation analysis was applied in the platform to assess the correlation of 20 numerical clinical indicators that showed significant differences (Figure 1C, the numbers in heatmap are correlation coefficients).

#### ROC analysis

ROC analysis of CHI3L1 was performed based on the cut-off values for diagnosing advanced liver fibrosis (F3 and F4, 78.85 ng/mL) and cirrhosis (F4, 107.70 ng/mL, Figure 1B).

Data presented as the median with interquartile range. The differences were considered significant if *p* < 0.05.

## References

[1] Kisseleva T., Brenner D. (2021). Molecular and cellular mechanisms of liver fibrosis and its regression. Nat. Rev. Gastroenterol. Hepatol..

[2] Bedossa P., Carrat F. (2009). Liver biopsy: the best, not the gold standard. J. Hepatol..

[3] Regev A., Berho M., Jeffers L.J., Milikowski C., Molina E.G., Pyrsopoulos N.T., Feng Z.Z., Reddy K.R., Schiff E.R. (2002). Sampling error and intraobserver variation in liver biopsy in patients with chronic HCV infection. Am. J. Gastroenterol..

[4] Tapper E.B., Loomba R. (2018). Noninvasive imaging biomarker assessment of liver fibrosis by elastography in NAFLD. Nat. Rev. Gastroenterol. Hepatol..

[5] Eddowes P.J., Sasso M., Allison M., Tsochatzis E., Anstee Q.M., Sheridan D., Guha I.N., Cobbold J.F., Deeks J.J., Paradis V. (2019). Accuracy of FibroScan Controlled Attenuation Parameter and Liver Stiffness Measurement in Assessing Steatosis and Fibrosis in Patients With Nonalcoholic Fatty Liver Disease. Gastroenterology.

[6] Park C.C., Nguyen P., Hernandez C., Bettencourt R., Ramirez K., Fortney L., Hooker J., Sy E., Savides M.T., Alquiraish M.H. (2017). Magnetic Resonance Elastography vs Transient Elastography in Detection of Fibrosis and Noninvasive Measurement of Steatosis in Patients With Biopsy-Proven Nonalcoholic Fatty Liver Disease. Gastroenterology.

[7] Friedrich-Rust M., Ong M.F., Martens S., Sarrazin C., Bojunga J., Zeuzem S., Herrmann E. (2008). Performance of transient elastography for the staging of liver fibrosis: a meta-analysis. Gastroenterology.

[8] Pavlov C.S., Casazza G., Nikolova D., Tsochatzis E., Burroughs A.K., Ivashkin V.T., Gluud C. (2015). Transient elastography for diagnosis of stages of hepatic fibrosis and cirrhosis in people with alcoholic liver disease. Cochrane Database Syst. Rev..

[9] Pavlov C.S., Casazza G., Nikolova D., Tsochatzis E., Gluud C. (2016). Systematic review with meta-analysis: diagnostic accuracy of transient elastography for staging of fibrosis in people with alcoholic liver disease. Aliment. Pharmacol. Ther..

[10] Smilowitz N.R., Feit F. (2016). The History of Primary Angioplasty and Stenting for Acute Myocardial Infarction. Curr. Cardiol. Rep..

[11] Selvaraj E.A., Mózes F.E., Jayaswal A.N.A., Zafarmand M.H., Vali Y., Lee J.A., Levick C.K., Young L.A.J., Palaniyappan N., Liu C.H. (2021). Diagnostic accuracy of elastography and magnetic resonance imaging in patients with NAFLD: A systematic review and meta-analysis. J. Hepatol..

[12] Nishimura N., De Battista D., McGivern D.R., Engle R.E., Tice A., Fares-Gusmao R., Kabat J., Pomerenke A., Nguyen H., Sato S. (2021). Chitinase 3-like 1 is a profibrogenic factor overexpressed in the aging liver and in patients with liver cirrhosis. USA.

[13] Kim J., Seki E. (2023). Hyaluronan in liver fibrosis: basic mechanisms, clinical implications, and therapeutic targets. Hepatol. Commun..

[14] Vali Y., Lee J., Boursier J., Spijker R., Löffler J., Verheij J., Brosnan M.J., Böcskei Z., Anstee Q.M., Bossuyt P.M. (2020). Enhanced liver fibrosis test for the non-invasive diagnosis of fibrosis in patients with NAFLD: A systematic review and meta-analysis. J. Hepatol..

[15] Kjaergaard M., Lindvig K.P., Thorhauge K.H., Andersen P., Hansen J.K., Kastrup N., Jensen J.M., Hansen C.D., Johansen S., Israelsen M. (2023). Using the ELF test, FIB-4 and NAFLD fibrosis score to screen the population for liver disease. J. Hepatol..

[16] Lichtinghagen R., Pietsch D., Bantel H., Manns M.P., Brand K., Bahr M.J. (2013). The Enhanced Liver Fibrosis (ELF) score: normal values, influence factors and proposed cut-off values. J. Hepatol..

[17] Leroy V., Monier F., Bottari S., Trocme C., Sturm N., Hilleret M.N., Morel F., Zarski J.P. (2004). Circulating matrix metalloproteinases 1, 2, 9 and their inhibitors TIMP-1 and TIMP-2 as serum markers of liver fibrosis in patients with chronic hepatitis C: comparison with PIIINP and hyaluronic acid. Am. J. Gastroenterol..

[18] Friedman S.L., Pinzani M. (2022). Hepatic fibrosis 2022: Unmet needs and a blueprint for the future. Hepatology.

[19] Cao L.L., Peng M., Xie X., Chen G.Q., Huang S.Y., Wang J.Y., Jiang F., Cui X.W., Dietrich C.F. (2022). Artificial intelligence in liver ultrasound. World J. Gastroenterol..

[20] D'Amico G., Morabito A., D'Amico M., Pasta L., Malizia G., Rebora P., Valsecchi M.G. (2018). Clinical states of cirrhosis and competing risks. J. Hepatol..

[21] Toh M.R., Wong E.Y.T., Wong S.H., Ng A.W.T., Loo L.H., Chow P.K.H., Ngeow J. (2023). Global Epidemiology and Genetics of Hepatocellular Carcinoma. Gastroenterology.

[22] Wong G.L.H., Yuen P.C., Ma A.J., Chan A.W.H., Leung H.H.W., Wong V.W.S. (2021). Artificial intelligence in prediction of non-alcoholic fatty liver disease and fibrosis. J. Gastroenterol. Hepatol..

[23] Hassoun S., Bruckmann C., Ciardullo S., Perseghin G., Marra F., Curto A., Arena U., Broccolo F., Di Gaudio F. (2024). NAIF: A novel artificial intelligence-based tool for accurate diagnosis of stage F3/F4 liver fibrosis in the general adult population, validated with three external datasets. Int. J. Med. Inf..

[24] Chen L.D., Huang Z.R., Yang H., Cheng M.Q., Hu H.T., Lu X.Z., Li M.D., Lu R.F., He D.N., Lin P. (2024). US-based Sequential Algorithm Integrating an AI Model for Advanced Liver Fibrosis Screening. Radiology.

[25] Zhang C., Shu Z., Chen S., Peng J., Zhao Y., Dai X., Li J., Zou X., Hu J., Huang H. (2024). A machine learning-based model analysis for serum markers of liver fibrosis in chronic hepatitis B patients. Sci. Rep..

[26] Sarvestany S.S., Kwong J.C., Azhie A., Dong V., Cerocchi O., Ali A.F., Karnam R.S., Kuriry H., Shengir M., Candido E. (2022). Development and validation of an ensemble machine learning framework for detection of all-cause advanced hepatic fibrosis: a retrospective cohort study. Lancet Digit. Health.

[27] Johansen J.S., Williamson M.K., Rice J.S., Price P.A. (1992). Identification of proteins secreted by human osteoblastic cells in culture. J. Bone Miner. Res..

[28] Lee C.G., Da Silva C.A., Dela Cruz C.S., Ahangari F., Ma B., Kang M.J., He C.H., Takyar S., Elias J.A. (2011). Role of chitin and chitinase/chitinase-like proteins in inflammation, tissue remodeling, and injury. Annu. Rev. Physiol..

[29] Liu W., Yang Y.J., An Q. (2020). LINC00963 Promotes Ovarian Cancer Proliferation, Migration and EMT via the miR-378g/CHI3L1 Axis. Cancer Manag. Res..

[30] Jin X., Fu B., Wu Z.J., Zheng X.Q., Hu J.H., Jin L.F., Tang L.L. (2020). Serum chitinase-3-like protein 1 is a biomarker of liver fibrosis in patients with chronic hepatitis B in China. Hepatobiliary Pancreat. Dis. Int..

[31] Bao J., Ouyang Y., Qiao L., He J., Liu F., Wang Y., Miao L., Fu A., Lou Z., Zang Q. (2022). Serum CHI3L1 as a Biomarker for Non-invasive Diagnosis of Liver Fibrosis. Discov. Med..

[32] Pungpapong S., Nunes D.P., Krishna M., Nakhleh R., Chambers K., Ghabril M., Dickson R.C., Hughes C.B., Steers J., Nguyen J.H., Keaveny A.P. (2008). Serum fibrosis markers can predict rapid fibrosis progression after liver transplantation for hepatitis C. Liver Transplant..

[33] Kamal S.M., Turner B., He Q., Rasenack J., Bianchi L., Al Tawil A., Nooman A., Massoud M., Koziel M.J., Afdhal N.H. (2006). Progression of fibrosis in hepatitis C with and without schistosomiasis: correlation with serum markers of fibrosis. Hepatology.

[34] Harrison S.A., Ratziu V., Boursier J., Francque S., Bedossa P., Majd Z., Cordonnier G., Sudrik F.B., Darteil R., Liebe R. (2020). A blood-based biomarker panel (NIS4) for non-invasive diagnosis of non-alcoholic steatohepatitis and liver fibrosis: a prospective derivation and global validation study. Lancet Gastroenterol. Hepatol..

[35] Kjaergaard A.D., Bojesen S.E., Nordestgaard B.G., Johansen J.S. (2014). YKL-40 and alcoholic liver and pancreas damage and disease in 86,258 individuals from the general population: cohort and mendelian randomization studies. Clin. Chem..

[36] Kim M.N., An J., Kim E.H., Kim H.Y., Lee H.A., Yu J.H., Jin Y.J., Chon Y.E., Kim S.U., Jun D.W. (2024). Vibration-controlled transient elastography for significant fibrosis in treatment-naive chronic hepatitis B patients: A systematic review and meta-analysis. Clin. Mol. Hepatol..

[37] Chon Y.E., Jin Y.J., An J., Kim H.Y., Choi M., Jun D.W., Kim M.N., Han J.W., Lee H.A., Yu J.H., Kim S.U. (2024). Optimal cut-offs of vibration-controlled transient elastography and magnetic resonance elastography in diagnosing advanced liver fibrosis in patients with nonalcoholic fatty liver disease: A systematic review and meta-analysis. Clin. Mol. Hepatol..

[38] An J., Chon Y.E., Kim G., Kim M.N., Kim H.Y., Lee H.A., Yu J.H., Choi M., Jun D.W., Kim S.U. (2024). Diagnostic accuracy of vibration-controlled transient elastography for staging liver fibrosis in autoimmune liver diseases: A systematic review and meta-analysis. Clin. Mol. Hepatol..

[39] Kim M.N., Han J.W., An J., Kim B.K., Jin Y.J., Kim S.S., Lee M., Lee H.A., Cho Y., Kim H.Y. (2024). KASL clinical practice guidelines for noninvasive tests to assess liver fibrosis in chronic liver disease. Clin. Mol. Hepatol..

