# Supplementary material for: Leveraging serum CHI3L1 and machine learning for non-invasive liver fibrosis diagnostics
Source: iScience. 2026 Aug 31;29(9):117372. doi: 10.1016/j.isci.2026.117372 (PMC13560028; doi:10.1016/j.isci.2026.117372)
Supplement: Supplementary file 1 [file mmc1.pdf]

iScience, Volume *29*

Supplemental information

Leveraging serum CHI3L1 and machine learning for non-invasive liver fibrosis diagnostics

Haizhen Chen, Gaixia Zhang, Yingwen Zhang, Li Li, Yuanyuan Che, and Jing Huang

Supplemental Figures and Legends

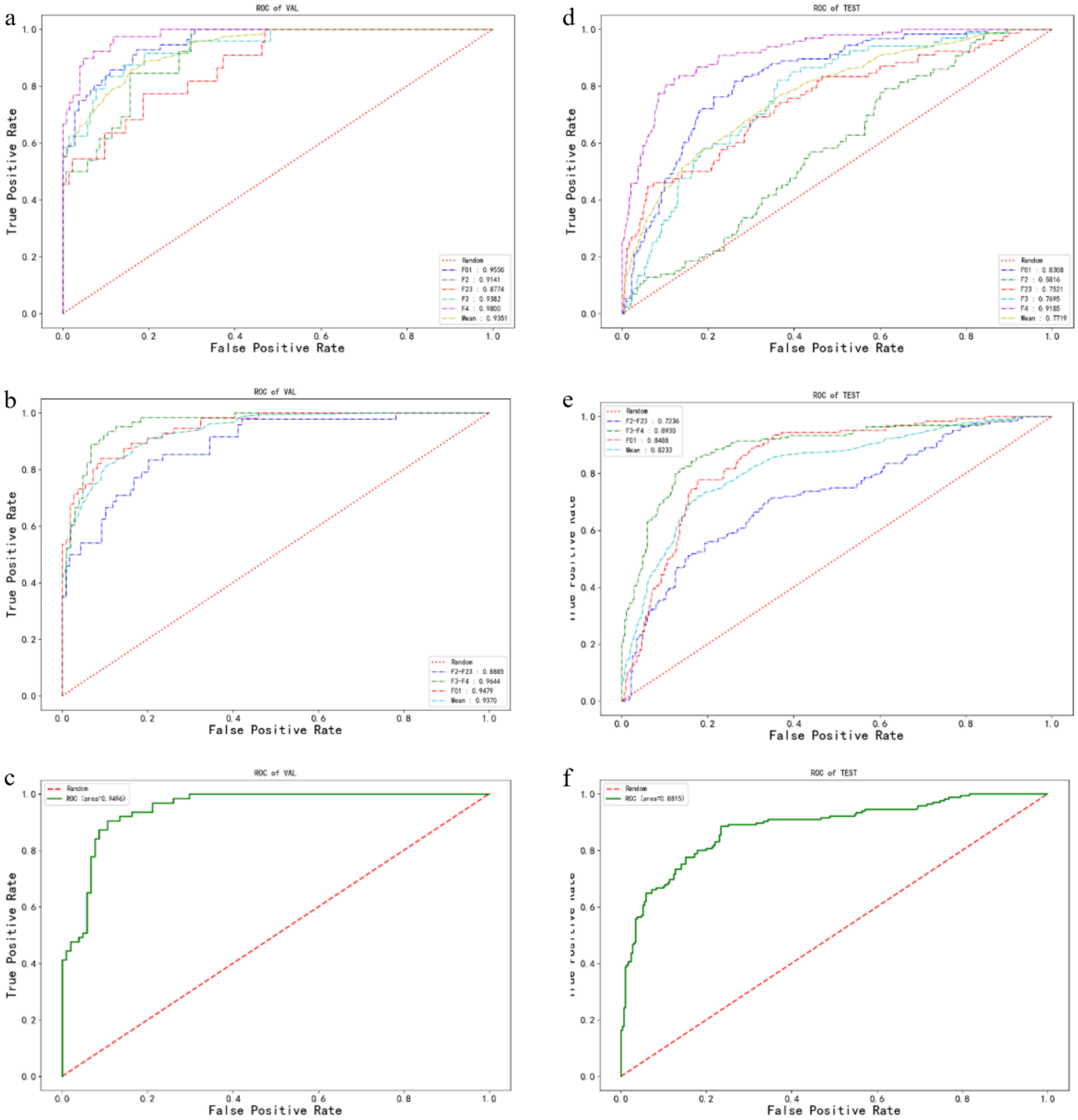

Figure S1. The ROC curve of XG-Boost liver fibrosis diagnostic model.

(a) The ROC curve of FIB5-7 XG-Boost model in training cohort.

(b) The ROC curve of FIB3-7 XG-Boost model in training cohort.

(c) The ROC curve of FIB2-7 XG-Boost model in training cohort.

(d) The ROC curve of FIB5-7 XG-Boost model in validation cohort.

(e) The ROC curve of FIB3-7 XG-Boost model in validation cohort.

The ROC curve of FIB2-7 XG-Boost model in validation cohort.

Supplemental Tables

Table S1. Spearman correlation analysis for differential variables of liver fibrosis.

	CHI3L1	PLT	MCH	HCT	HB	RBC	TBA	IBIL	DBIL	TBIL	A/G	GLO	ALB	TP	CHE	ALP	GGT	ALT	AST	Age
CHI3L1	1.00	**-0.37**	**0.27**	**-0.29**	**-0.29**	**-0.38**	**0.50**	**0.20**	**0.31**	**0.22**	**-0.43**	**0.31**	**-0.44**	-0.01	**-0.48**	**0.37**	**0.33**	0.00	**0.28**	**0.43**
PLT	**-0.37**	1.00	**-0.18**	**0.18**	**0.11**	**0.22**	**-0.48**	**-0.30**	**-0.42**	**-0.32**	**0.20**	-0.03	**0.33**	**0.24**	**0.47**	**-0.23**	**-0.19**	-0.06	**-0.26**	**-0.28**
MCH	**0.27**	**-0.18**	1.00	0.08	**0.20**	**-0.22**	**0.18**	**0.21**	**0.20**	**0.20**	**0.07**	**-0.13**	**-0.14**	**-0.25**	**-0.10**	**0.09**	**0.26**	**0.06**	**0.13**	**0.24**
HCT	**-0.29**	**0.18**	0.08	1.00	**0.95**	**0.91**	**-0.28**	**0.21**	0.06	**0.19**	**0.42**	**-0.18**	**0.53**	**0.22**	**0.50**	**-0.16**	**0.11**	**0.24**	0.00	**-0.30**
HB	**-0.29**	**0.11**	**0.20**	**0.95**	1.00	**0.86**	**-0.26**	**0.25**	**0.10**	**0.23**	**0.46**	**-0.24**	**0.52**	**0.15**	**0.47**	**-0.16**	0.10	**0.24**	0.01	**-0.28**
RBC	**-0.38**	**0.22**	**-0.22**	**0.91**	**0.86**	1.00	**-0.37**	**0.13**	**-0.02**	**0.11**	**0.42**	**-0.16**	**0.59**	**0.29**	**0.54**	**-0.20**	-0.01	**0.22**	-0.05	-0.40
TBA	**0.50**	**-0.48**	**0.18**	**-0.28**	**-0.26**	**-0.37**	1.00	**0.27**	**0.47**	**0.33**	**-0.39**	**0.20**	**-0.49**	**-0.16**	**-0.56**	**0.41**	**0.39**	**0.15**	**0.46**	**0.32**
IBIL	**0.20**	**-0.30**	**0.21**	**0.21**	**0.25**	**0.13**	**0.27**	1.00	**0.84**	**0.97**	-0.08	**0.12**	**0.03**	**0.11**	**-0.13**	**0.20**	**0.31**	**0.24**	**0.30**	0.05
DBIL	**0.31**	**-0.42**	**0.20**	0.06	**0.10**	**-0.02**	**0.47**	**0.84**	1.00	**0.89**	**-0.25**	**0.19**	**-0.21**	0.01	**-0.38**	**0.29**	**0.47**	**0.33**	**0.44**	0.07
TBIL	**0.22**	**-0.32**	**0.20**	**0.19**	**0.23**	**0.11**	**0.33**	**0.97**	**0.89**	1.00	**-0.12**	**0.14**	-0.02	0.10	**-0.19**	**0.24**	**0.36**	**0.30**	**0.34**	0.04
A/G	**-0.43**	**0.20**	**0.07**	**0.42**	**0.46**	**0.42**	**-0.39**	-0.08	**-0.25**	**-0.12**	1.00	**-0.86**	**0.67**	**-0.32**	**0.46**	**-0.34**	**-0.16**	-0.01	**-0.27**	**-0.29**
GLO	**0.31**	-0.03	**-0.13**	**-0.18**	**-0.24**	**-0.16**	**0.20**	**0.12**	**0.19**	**0.14**	**-0.86**	1.00	**-0.28**	**0.72**	**-0.20**	**0.28**	**0.11**	**0.05**	**0.25**	**0.14**
ALB	**-0.44**	**0.33**	**-0.14**	**0.53**	**0.52**	**0.59**	**-0.49**	**0.03**	**-0.21**	-0.02	**0.67**	**-0.28**	1.00	**0.39**	**0.61**	**-0.29**	**-0.12**	0.06	**-0.18**	**-0.36**
TP	-0.01	**0.24**	**-0.25**	**0.22**	**0.15**	**0.29**	**-0.16**	**0.11**	0.01	0.10	**-0.32**	**0.72**	**0.39**	1.00	**0.26**	0.08	0.01	0.08	**0.09**	**-0.10**
CHE	**-0.48**	**0.47**	**-0.10**	**0.50**	**0.47**	**0.54**	**-0.56**	**-0.13**	**-0.38**	**-0.19**	**0.46**	**-0.20**	**0.61**	**0.26**	1.00	**-0.34**	**-0.22**	0.03	**-0.23**	**-0.33**
ALP	**0.37**	**-0.23**	**0.09**	**-0.16**	**-0.16**	**-0.20**	**0.41**	**0.20**	**0.29**	**0.24**	**-0.34**	**0.28**	**-0.29**	0.08	**-0.34**	1.00	**0.40**	**0.29**	**0.39**	**0.18**
GGT	**0.33**	**-0.19**	**0.26**	**0.11**	0.10	-0.01	**0.39**	**0.31**	**0.47**	**0.36**	**-0.16**	**0.11**	**-0.12**	0.01	**-0.22**	**0.40**	1.00	**0.58**	**0.60**	0.04
ALT	0.00	-0.06	**0.06**	**0.24**	**0.24**	**0.22**	**0.15**	**0.24**	**0.33**	**0.30**	-0.01	**0.05**	0.06	0.08	0.03	**0.29**	**0.58**	1.00	**0.78**	**-0.17**
AST	**0.28**	**-0.26**	**0.13**	0.00	0.01	-0.05	**0.46**	**0.30**	**0.44**	**0.34**	**-0.27**	**0.25**	**-0.18**	**0.09**	**-0.23**	**0.39**	**0.60**	**0.78**	1.00	0.05
Age	**0.43**	**-0.28**	**0.24**	**-0.30**	**-0.28**	-0.40	**0.32**	0.05	0.07	0.04	**-0.29**	**0.14**	**-0.36**	**-0.10**	**-0.33**	**0.18**	0.04	**-0.17**	0.05	1.00

20 numerical variables were analyzed by spearman correlation analysis. The number is correlation coefficient and bold indicates $P < 0.05$. AST, aspartate aminotransferase; ALT, alanine aminotransferase; γGGT, gamma-glutamyl transferase; ALP, alkaline phosphatase; CHE, cholinesterase; TP, total protein; A, albumin; G, globulin; A/G, ratio of albumin and globulin; TBIL, total bilirubin; DBIL, direct bilirubin; IBIL, indirect bilirubin; TBA, total bile acids; RBC, red blood cell count; HB, hemoglobin; HCT, hematocrit; MCH, mean corpuscular hemoglobin; PLT, platelet; CHI3L1, chitinase 3 like 1.

Table S2. Comparison of the XG-Boost model FIB5-7 and FIB5-15.

Characteristics	AUC	Standard error	95% CI	z	*P* value
FIB5-7-F01	0.96	0.014	0.93-0.98	1.404	0.160
FIB5-15-F01	0.94	0.016	0.91-0.98		
FIB5-7-F2	0.91	0.024	0.87-0.96	1.732	0.083
FIB5-15-F2	0.87	0.037	0.80-0.94		
FIB5-7-F23	0.88	0.037	0.80-0.95	0.149	0.882
FIB5-15-F23	0.88	0.040	0.80-0.95		
FIB5-7-F34	0.94	0.025	0.89-0.99	-1.758	0.079
FIB5-15-F34	0.97	0.014	0.94-0.99		
FIB5-7-F4	0.98	0.009	0.96-0.99	0.394	0.693
FIB5-15-F4	0.98	0.011	0.96-1.00		

The *DeLong* test was used to analyze the differences between the FIB5-7 and FIB5-15 models. Area under roc curve, AUC; 95% confidence interval, 95% CI.

Table S3. Baseline clinical characteristics of validation cohort.

Baseline features	Validation cohort						
	F01 (n = 126)	F2 (n = 86)	F23 (n = 78)	F3 (n = 67)	F4 (n = 98)	Overall (n = 455)	*P*-value
Sex							
Female	58 (46.0)	36 (41.9)	37 (47.4)	24 (35.8)	38 (39.8)	193 (42.4)	0.567
Male	68 (54.0)	50 (58.1)	41 (52.6)	43 (64.2)	60 (60.2)	262 (57.6)	
Age (year)	52 (40–58)	47 (37–55)	51 (36–55)	53 (43–58)	53 (44–61)	53 (46–59)	<0.001
BMI (Kg/m^2)	24.5 (21.6–27.3)	23.4 (21.0–25.4)	24.8 (23.4–28.4)	25.0 (21.6–28.2)	24.5 (21.7–27.8)	24.2 (22.1–28.0)	0.002
AST (U/L)	29.9 (23.7–41.4)	25.2 (21.7–31.4)	28.5 (23.6–36.4)	30.9 (25.8–41.0)	33.0 (27.0–45.1)	41.2 (28.7–67.6)	<0.001
ALT (U/L)	26.8 (19.3–41.5)	23.0 (17.3–36.8)	27.1 (18.9–40.6)	32.2 (22.9–47.4)	26.8 (20.0–40.3)	31.8 (22.6–47.5)	0.004
γGGT (U/L)	36.7 (22.2–68.9)	23.4 (16.5–46.1)	33.4 (21.8–51.3)	32.2 (23.3–50.3)	42.4 (30.4–73.4)	62.6 (36.7–144.9)	0.007
ALP (U/L)	89.1 (70.1–114.1)	77.1 (65.1–96.8)	78.6 (64.3–101.4)	85.5 (68.9–112.2)	94.2 (76.7–114.4)	116.8 (90.0–175.2)	<0.001
CHE (U/L)	8012.5 (6267–9524)	8707 (7680–10144)	8786 (7565–10614)	8380 (7340–9520)	7170 (5778–8857)	4881 (3372–6633)	<0.001
TP (g/L)	75.7 (72.2–78.5)	75.7 (73.5–78.2)	76.7 (72.9–79.1)	76.1 (74.0–79.0)	74.4 (71.4–78.3)	74.1 (67.0–77.7)	0.007
ALB (g/L)	44.3 (41.7–46.4)	45.6 (44.1–46.9)	45.4 (43.4–46.4)	44.7 (42.9–46.1)	43.8 (42.1–45.9)	38.0 (33.0–42.7)	<0.001
GLO (g/L)	31.5 (28.6–34.6)	30.1 (28.2–32.2)	31.0 (28.1–34.2)	32.0 (29.5–34.0)	31.3 (28.3–35.3)	34.7 (31.1–38.5)	<0.001
A/G	1.41 (1.23–1.57)	1.54 (1.40–1.67)	1.48 (1.34–1.63)	1.39 (1.28–1.51)	1.39 (1.24–1.59)	1.10 (0.90–1.28)	0.004
TBIL (μmol/L)	16.3 (12.7–22.8)	14.2 (11.4–17.6)	14.5 (12.3–18.8)	16.0 (12.7–21.2)	16.8 (14.1–23.0)	24.5 (16.6–39.9)	<0.001
DBIL (μmol/L)	3.0 (2.3–4.4)	2.4 (2.0–3.2)	2.6 (2.1–3.6)	2.9 (2.2–4.2)	3.3 (2.8–4.9)	5.3 (3.6–12.6)	<0.001
IBIL (μmol/L)	13.3 (10.3–18.2)	11.7 (9.4–14.6)	11.8 (10.1–15.1)	13.0 (10.4– 17.6)	13.6 (11.0–18.5)	18.9 (13.5–28.6)	<0.001
TBA (μmol/L)	3.3 (1.90–8.9)	2.0 (1.5–3.0)	2.7 (1.8–4.)	3.1 (2.2 –6.2)	4.8 (3.0 –15.3)	17.9 (6.8 –43.8)	<0.001
RBC (10^{12}/L)	5.03 (4.61–5.14)	5.04 (4.84 –5.10)	5.03 (4.98–5.15)	5.14 (5.07–5.20)	4.93 (4.68–5.06)	4.28 (3.55–5.07)	<0.001
HB (g/L)	150 (136–162)	150 (141–162)	154 (142-164)	154 (146–161)	151 (137–163)	133 (112–160)	<0.001
HCT	0.44 (0.41–0.48)	0.45 (0.42–0.48)	0.46 (0.42–0.48)	0.46 (0.43–0.48)	0.45 (0.42–0.48)	0.39 (0.33–0.47)	<0.001
MCV (fL)	91.4 (88.–94.8)	90.6 (87.7–94.2)	90.0 (87.5–93.3)	90.9 (87.5–94.3)	93.0 (90.7–95.6)	92.6 (89.28–98.1)	0.001

MCH (pg)	31.0 (29.7–32.2)	30.9 (29.7–31.7)	30.3 (29.4–31.8)	30.7 (29.5–31.7)	31.5 (30.6–32.4)	31.3 (30.1–33.3)	0.01
PLT (10^9/L)	179 (134–223)	223 (212–238)	179 (173–187)	188 (179–204)	134 (123–143)	88 (71–123)	0.001
CHI3L1 (ng/mL)	66.0 (42.6–128.4)	50.4 (36.3–73.3)	51.6 (38.5–80.1)	57.4 (45.6–107.1)	78.3 (51.7–138.2)	162.0 (95.2–309.1)	0.01

The classification data of clinical characteristics is represented as the number (percentage), and the quantitative data is represented as median (interquartile range). In the first row of the table, "n" represents the number of participants in the liver fibrosis group. The *Pearson's Chi-squared* test was used for difference of sex between groups, and the *Kruskal-Wallis* rank sum test was used for other baseline features. BMI, body mass index; AST, aspartate aminotransferase; ALT, alanine aminotransferase; γGGT, gamma-glutamyl transferase; ALP, alkaline phosphatase; CHE, cholinesterase; PAB, prealbumin; TP, total protein; A, albumin; G, globulin; A/G, ratio of albumin and globulin; TBIL, total bilirubin; DBIL, direct bilirubin; IBIL, indirect bilirubin; TBA, total bile acids; RBC, red blood cell count; HB, hemoglobin; HCT, hematocrit; MCV, mean corpuscular volume; MCH, mean corpuscular hemoglobin; PLT, platelet; CHI3L1, chitinase 3 like 1.

Table S4. The FIB5-7 model of XG-Boost in validation cohort.

Fibrosis stage	AUC	F1 score	Sensitivity	Specificity	Accuracy	NPV	PPV
F01	0.83	0.64	0.84	0.70	0.74	0.92	0.52
F2	0.58	0.13	0.08	0.95	0.78	0.82	0.27
F23	0.75	0.46	0.47	0.88	0.81	0.89	0.45
F34	0.76	0.39	0.39	0.90	0.82	0.89	0.40
F4	0.92	0.66	0.59	0.94	0.87	0.89	0.74
Mean	0.77	0.46	0.48	0.87	0.51	0.88	0.48

AUC, area under roc curve; NPV, positive predictive value; NPV, negative predictive value.

Table S5. The FIB3-7 model of XG-Boost in validation cohort.

Fibrosis stage	AUC	F1 score	Sensitivity	Specificity	Accuracy	NPV	PPV
F01	0.72	0.59	0.58	0.79	0.71	0.77	0.61
F2 & F23	0.89	0.77	0.75	0.88	0.83	0.86	0.78
F34 & F4	0.85	0.66	0.70	0.85	0.80	0.88	0.63
Mean	0.82	0.67	0.68	0.84	0.67	0.84	0.67

AUC, area under roc curve; NPV, positive predictive value; NPV, negative predictive value.

Table S6. The FIB2-7 model of XG-Boost in validation cohort.

Positive stage	AUC	F1 score	Sensitivity	Specificity	Accuracy	NPV	PPV
F3-F4	0.88 (0.85-0.92)	0.74	0.67	0.83	0.83	0.83	0.82

AUC, area under roc curve; NPV, positive predictive value; NPV, negative predictive value.

Data S1/Methods S1: Supplementary statistical protocols, transient elastography examination procedures, and clinical laboratory data processing for the serum CHI3L1-based machine-learning model for non-invasive liver fibrosis diagnosis.

1. Statistical Analysis:

This study utilized the Extreme Smart Analysis platform (https://www.xsmartanalysis.com/) built on R Studio software for data analysis. Study subjects were independently allocated into the training cohort and external validation cohort based on time series. Count data were presented as numbers and proportions, with chi-square tests or Fisher's exact probability tests used when expected frequencies were <10. Normal distribution measurement data were expressed as mean ± standard deviation, and intergroup comparisons were performed using t-tests. Non-normal distribution continuous variables were expressed as median and interquartile range, with intergroup comparisons conducted using the *Mann-Whitney U* test. The significance level was set at $\alpha = 0.05$.

In the training cohort, intergroup difference analysis was employed to screen for differential indicators of liver fibrosis staging from the initial 23 variables. Subsequently, Spearman correlation analysis was performed on all differential indicators to eliminate strongly correlated parameters and avoid overfitting in the liver fibrosis diagnostic model. Ultimately, 15 liver fibrosis-specific and independently correlated indicators were selected as initial parameters. Five machine learning algorithms - Adaboost, XGBoost, Random Forest, Gradient Boosting, and Decision Tree - were used to construct a five-class (F01, F2, F23, F3, and F4 stages) liver fibrosis diagnostic model through 3- to 7-fold cross-validation method. The optimal model was selected based on evaluation metrics such as the area under the receiver operating characteristic (ROC) curve, specificity, precision, sensitivity, and F1 score in the training cohort. The 3- to 7-fold cross-validation enabled internal validation of the model, preliminarily optimizing the diagnostic model established from the training cohort. A diagnostic model was rebuilt using the top 7 parameters with relatively high weights (> 0.35) in the best-performing model. Subsequently, the DeLong test was used to compare the diagnostic performance between the 15-parameter and 7-parameter models. If no significant difference was observed, the simplified 7-parameter model could replace the 15-parameter model. Liver fibrosis groups with low sensitivity were merged to further optimize the selected model. The above diagnostic models were then validated in the external validation cohort, where performance was similarly assessed using the area under the ROC curve, specificity, precision, sensitivity, and F1 score. The R-language functionality within the machine learning platform was employed to generate calibration curves and decision curve analysis (DCA) plots to

evaluate the clinical applicability of the models. Finally, the DeLong test was used to compare the diagnostic performance of the established liver fibrosis models with that of APRI and FIB-4.

2. Standardized Transient Elastography (TE) Examination Protocol:

(1). Operator qualifications:

- Examiners must demonstrate proficiency with ≥500 prior TE procedures before performing independent examinations.

(2). Patient preparation:

- Fasting requirement: 2-3 hours prior to examination;
- Pre-procedural rest: 10-20 minutes in supine position.

(3). Positioning technique:

- Patient positioning: Supine or slight left lateral decubitus position;
- Right arm placement: Elevated above the head;
- Probe application: Perpendicular to skin surface with moderate pressure at intercostal space.

(4). Measurement protocol:

- Respiratory phase: Mid-expiration breath hold (3-5 seconds);
- Sampling depth: 1-2 cm beneath liver capsule (maximum 6 cm depth);
- Image acquisition: Continuous monitoring until stable elastography images are obtained.

(5). Anatomical Avoidance Criteria:

- Prohibited measurement zones: rib shadows, vascular/biliary structures (diameter >2 mm), gas-containing structures;
- Acquisition timing: Avoid deep inspiration breath-holding.

(6). Quality Control:

- Image stability: Requires ≥5 consecutive consistent measurements;
- Sampling window: Must demonstrate homogeneous parenchymal texture.

(7). Diagnostic criteria:

- liver stiffness value (LSM) < 7.3 kPa: F0-F1 stage, no fibrosis or mild fibrosis;
- LSM 7.3-9.7 kPa: F2, mild fibrosis;
- LSM 9.7-12.4 kPa: F2-F3, moderate fibrosis;
- LSM 12.4-17.5 kPa: F3-F4, severe fibrosis;
- LSM > 17.5 kPa: liver cirrhosis.

3. Clinical laboratory data acquisition and analysis

All laboratory parameters were obtained from the electronic information system of the Department of Laboratory Medicine at the First Hospital of Jilin University. Detailed methodological procedures and instrument specifications are provided in the table below. In this study, APRI and FIB-4 serological were used to compare with FIB2-7 fibrosis diagnosis model, and mixed model formulas can be follows:

$$APRI = \frac{\mathrm{AST(IU/L)/ULN((upper\ limit\ of\ mormal))}}{\mathrm{PLT(10^9/L)}} \times 100$$

$$FIB-4 = \frac{\mathrm{Age(year)} \times \mathrm{AST(IU/L)}}{\mathrm{PLT(10^9/L)} \times \sqrt{\mathrm{ALT(IU/L)}}} \times 100$$

Laboratory parameters testing instrument

Features	Instrument manufacturing company	Instrument model
Transient elastography	Echosens	FibroScan 502
CHI3L1 level	YHLO Corporation	iFlash
Blood routine indexes	Sysmex Corporation	Sysmex XN-9000
Blood biochemical indexes	Beckman Coulter, Inc	AU5800
Coagulation function indexes	Sysmex Corporation	Sysmex cs-5100
Tumor markers	Roche Corporation	Cobas e601
